# Conversion to Everolimus was Beneficial and Safe for Fast and Slow Tacrolimus Metabolizers after Renal Transplantation

**DOI:** 10.3390/jcm9020328

**Published:** 2020-01-23

**Authors:** Gerold Thölking, Nils Hendrik Gillhaus, Katharina Schütte-Nütgen, Hermann Pavenstädt, Raphael Koch, Barbara Suwelack, Stefan Reuter

**Affiliations:** 1 Department of Internal Medicine and Nephrology, University Hospital of Münster Marienhospital Steinfurt, 48565 Steinfurt, Germany; 2 Department of Medicine D, Division of General Internal Medicine, Nephrology and Rheumatology, University Hospital of Münster, 48149 Münster, Germany; nilshendrik.gillhaus@ukmuenster.de (N.H.G.); katharina.schuette-nuetgen@ukmuenster.de (K.S.-N.); hermann.pavenstaedt@ukmuenster.de (H.P.); barbara.suwelack@ukmuenster.de (B.S.); stefan.reuter@ukmuenster.de (S.R.); 3 Institute of Biostatistics and Clinical Research, University of Münster, 48149 Münster, Germany; raphael.koch@ukmuenster.de

**Keywords:** tacrolimus, C/D ratio, tacrolimus metabolism, everolimus, conversion, kidney transplantation

## Abstract

Fast tacrolimus (TAC) metabolism (concentration/dose (C/D) ratio <1.05 ng/mL/mg) is a risk factor for inferior outcomes after renal transplantation (RTx) as it fosters, e.g., TAC-related nephrotoxicity. TAC minimization or conversion to calcineurin-inhibitor free immunosuppression are strategies to improve graft function. Hence, we hypothesized that especially patients with a low C/D ratio profit from a switch to everolimus (EVR). We analyzed data of 34 RTx recipients (17 patients with a C/D ratio <1.05 ng/mL/mg vs. 17 patients with a C/D ratio ≥1.05 ng/mL/mg) who were converted to EVR within 24 months after RTx. The initial immunosuppression consisted of TAC, mycophenolate, prednisolone, and basiliximab induction. During an observation time of 36 months after changing immunosuppression from TAC to EVR, renal function, laboratory values, and adverse effects were compared between the groups. Fast TAC metabolizers were switched to EVR 4.6 (1.5–21.9) months and slow metabolizers 3.3 (1.8–23.0) months after RTx (*p* = 0.838). Estimated glomerular filtration rate (eGFR) did not differ between the groups at the time of conversion (baseline). Thereafter, the eGFR in all patients increased noticeably (fast metabolizers eGFR 36 months: + 11.0 ± 11.7 (*p* = 0.005); and slow metabolizers eGFR 36 months: + 9.4 ± 15.9 mL/min/1.73 m^2^ (*p* = 0.049)) vs. baseline. Adverse events were not different between the groups. After the switch, eGFR values of all patients increased statistically noticeably with a tendency towards a higher increase in fast TAC metabolizers. Since conversion to EVR was safe in a three-year follow-up for slow and fast TAC metabolizers, this could be an option to protect fast metabolizers from TAC-related issues.

## 1. Introduction

Tarolimus (TAC)-based therapy is the recommended immunosuppressive standard therapy after renal transplantation (RTx), although its numerous adverse effects include the development of acute and chronic nephrotoxicity [1]. Unfortunately, TAC has a narrow therapeutic window and a high inter- and intraindividual variable pharmacokinetics, which requires therapeutic drug monitoring (TDM). TAC metabolism is subject to several non-modifiable factors such as age, sex, and CYP3A4/5 genotype of the RTx recipient as well as parameters that may vary, e.g., hematocrit, serum albumin, and steroid doses [2]. In view of the variety of impacting factors, transplant physicians are waiting for a stratification method to identify individuals with a high risk to develop TAC-related adverse effects.

Recently, we and others described a simple and cost-effective tool, the TAC concentration/dose ratio (C/D ratio), to address this problem [3,4]. The C/D ratio is calculated by dividing the TAC trough level by the daily TAC dose. To keep the tool as simple and practical as possible for clinical application, we decided to use only two different C/D ratio categories, although our first approach involved three [4]. A TAC C/D ratio < 1.05 ng/mL/mg assessed three months after RTx indicates fast TAC metabolism, whereas a C/D ratio ≥ 1.05 ng/mL/mg is suggestive of individuals with slow Tac clearance [5]. Using this C/D ratio cut off, we and others showed that the renal function of fast metabolizers is inferior to that of slow metabolizers after RTx and liver transplantation (cut off 1.09 ng/mL/mg), which is due to, e.g., higher incidences of TAC-related nephrotoxicity and rejections [4,5,6,7,8,9,10]. This resulted in decreased graft and patient survival [5,7]. In view of the data, modifications of the immunosuppressive regime of patients with a C/D ratio < 1.05 ng/mL/mg should be considered.

The ZEUS study showed that conversion of RTx recipients from calcineurin inhibitor (CNI) to everolimus (EVR) 4.5 months posttransplant is associated with a significant improvement in renal function, which is maintained for at least five years after RTx [11]. Despite increased rates of early mild acute rejections, long-term graft function was not affected in patients who switched to EVR. A positive effect of conversion from CNI to EVR on renal function was even shown for late conversion after RTx (after a mean of 82.6 months) [12]. However, in none of these studies was a C/D ratio-based stratification investigated in this regard.

Due to the negative impact of TAC on the outcomes of fast metabolizers, we hypothesized that these patients, after conversion to EVR, might have greater benefits than slow metabolizers.

## 2. Patients and Methods

### 2.1. Patients

This retrospective study included 17 fast metabolizers and 17 slow metabolizers undergoing RTx at the University Hospital of Münster, Germany, between December 2007 and November 2013. The inclusion criteria comprised: age ≥ 18 years of age, intake of immediate release TAC since RTx, and switch from TAC to EVR within 24 months after RTx. All patients received an initial immunosuppression with TAC (Prograf®), mycophenolate mofetil (CellCept®), prednisolone (Decortin H®/Soludecortin H®), and an induction therapy with basiliximab (Simulect®) at Days 0 and 4. TAC target trough levels were 7–12 ng/mL until the end of Month 1, 6–10 ng/mL for Months 2–3, and 3–8 ng/mL subsequently. The starting dose of mycophenolate mofetil 1 g twice a day (b.i.d). was adjusted in case of adverse effects. Prednisolone was started with 250 mg before and directly after RTx and tapered to a maintenance dosage of 5 mg once daily (q.d.) after six months. The recipient’s data were taken from the electronic health records of the hospital information system. Patients were switched from TAC to EVR with a target trough level of 3–8 ng/mL.

Renal function and complications were observed in a 36-month follow-up after conversion to EVR. Renal function was expressed as the estimated glomerular filtration rate (eGFR) calculated by the CKD-EPI formula [13]. Creatinine was analyzed in a whole blood sample (enzymatic assay; Creatinine-Pap, Roche Diagnostics, Mannheim, Germany). Proteinuria was assessed using spot urine. TAC levels were determined using the automated tacrolimus (TACR) assay (Dimension Clinical Chemistry System, Siemens Healthcare Diagnostic GmbH, Eschborn, Germany). EVR levels were measured by LC-MS/MS. Only 12-h TAC and EVR trough levels were used for analysis. Donor-specific antibodies (DSA) were assessed by single beat antigen assay (Luminex).

The C/D ratio was calculated using the following formula:

(1) $$C/D\;ratio\;\;\left(\mathrm{ng}/\mathrm{mL}*1/\mathrm{mg}\right)\;=\;\frac{blood\;TAC\;trough\;level\;\left(\mathrm{ng}/\mathrm{mL}\right)}{daily\;TAC\;dose\;\left(\mathrm{mg}\right)}$$

The TAC C/D ratio was calculated one month after RTx and used for grouping [14]. RTx recipients with a C/D ratio <1.05 ng/mL/mg were defined as fast and with a C/D ratio ≥1.05 ng/mL/mg as slow metabolizers.

Histologic results on rejections were obtained only from indication biopsies. All biopsy specimens had been reviewed by two pathologists in the local Institute of Pathology according to the revised Banff criteria [15].

The data of all RTx recipients were anonymized prior to analysis. The study was approved by the local ethics committee (Ethik Kommission der Ärztekammer Westfalen-Lippe und der Medizinischen Fakultät der Westfälischen Wilhelms-Universität, No. 2014-381-f-N). All participants in this study had given written informed consent to record their clinical data and to use it in anonymized analyses at the time of transplantation.

### 2.2. Statistical Analyses

IBM SPSS Statistics 26 for Windows (IBM Corporation, Somers, NY, USA) were used for statistical analyses of all data. All *p*-values were two-sided and were intended to be exploratory, not confirmatory. Exploratory *p*-values ≤0.05 were denoted as statistically noticeable. Absolute and relative frequencies are given for categorical variables. Normally-distributed continuous variables are shown as mean ± standard deviation and not normally-distributed continuous variables as median (minimum–maximum). The corresponding pairwise comparisons between fast and slow metabolizers were performed using Welch’s t-tests for normally distributed data, exact Mann–Whitney U tests for skewed distributed continuous variables, and Fisher’s exact tests for categorical variables without adjusting for multiple testing. Intra-group changes between two points in time were analyzed using Wilcoxon signed-rank tests for related samples. Boxplots were used for graphical representation.

## 3. Results

### 3.1. Descriptive Statistics

Patient characteristics, transplantation data, and immunosuppression after the first month are given in Table 1. Slow metabolizers tended to be older and had a lighter body weight, but all characteristics did not differ noticeably between groups. Fast metabolizers were converted from TAC to EVR after a median of 4.6 (1.5–21.9) months, slow metabolizers 3.3 (1.8–23.0) months after RTx (*p* = 0.832). Despite similar TAC trough levels after the first month (M1), TAC doses were noticeably higher and C/D ratio values were lower for fast metabolizers than for slow metabolizers (both *p* < 0.001), due to group classification.

The main reason for a conversion from TAC to EVR was CNI-nephrotoxicity in both metabolism groups (Table 2).

### 3.2. Renal Function 

**Figure 1:**
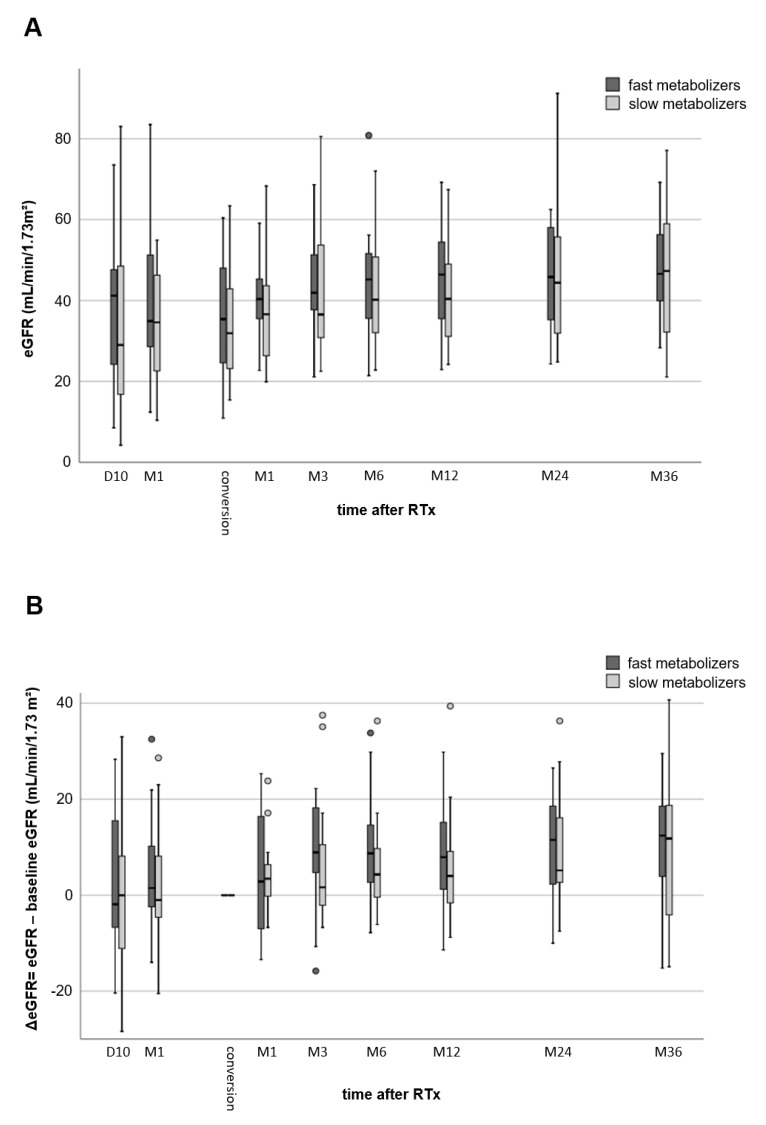
Comparison of renal function (eGFR values) of fast and slow TAC metabolizers. Both groups showed a considerable increase in renal function from Day 10 after kidney transplantation to 36 months after conversion from TAC to EVR (no differences between the groups) (**A**). Comparison of eGFR values to baseline eGFR (time of conversion from TAC to EVR) (**B**). Thirty-six months after transplantation, renal function of slow metabolizers showed a noticeable increase (*p* = 0.049), while fast metabolizers a highly noticeable increase (*p* = 0.005).

The renal function of fast and slow metabolizers was similar ten days after RTx (39.2 ± 19.7 vs. 33.7 ± 22.5 mL/min/1.73 m^2^, *p* = 0.456), one month after RTx (39.4 ± 18.8 vs. 34.2 ± 13.5 mL/min/1.73 m^2^, *p* = 0.367), and at the time of conversion of TAC to EVR (35.1 ± 15.2 vs. 34.2 ± 13.2 mL/min/1.73 m^2^, *p* = 0.850, Figure 1A). Figure 1B provides the renal function at different time points minus the baseline eGFR (eGFR at the time of conversion, Month 0 (M0)). At the end of the follow-up, the eGFR of the fast TAC metabolizers increased considerably by 11.0 ± 11.7 mL/min/1.73 m^2^ (*p* = 0.005, Figure 1B) compared to 9.4 ± 15.9 mL/min/1.73 m^2^ in slow metabolizers (*p* = 0.049). These changes were not statistically noticeably different between both groups (*p* = 0.691), but more homogenous in fast metabolizers.

### 3.3. Adverse Events

**Figure 2:**
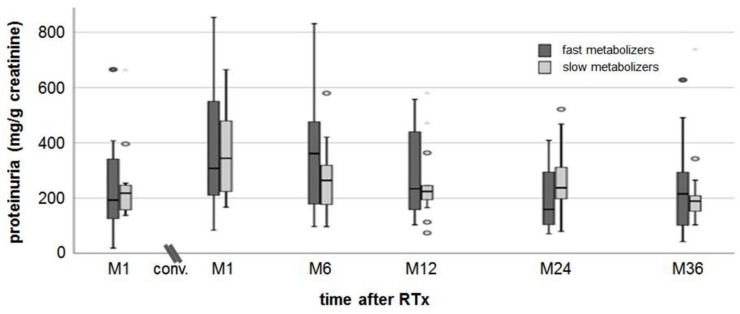
Proteinuria. There was a slight increase in proteinuria in both groups from M1 after RTx to M1 after conversion. At a follow-up of 36 months post-conversion, proteinuria recovered to values measured at M1 after RTx.

The median proteinuria value of fast metabolizers was 193 (19–665) mg/g creatinine at M1 after RTx and 361 (97–831) mg/g creatinine at M6 (maximum values) after conversion (Figure 2). The proteinuria in slow metabolizers was 218 (137–664) mg/g creatinine at M1 after RTx and 344 (167–665) mg/g creatinine at M6 (maximum values). At M36, proteinuria had declined to the baseline values without difference between the groups at all time points.

Table 3 shows the adverse events before and after conversion to EVR. There was no graft loss and no differences in outcomes such as delayed graft function (DGF) or overall survival between the groups. The DSA number in all patient groups before and after conversion was low and did not change noticeably. Although it was 9 vs. 6 biopsy-proven acute rejection (BPAR) cases in fast vs. slow metabolizers before conversion to EVR, BPAR rates were considerably lower during follow-up (two episodes (12%) in fast metabolizers and one episode (6%) in slow metabolizer) than before conversion. Cytomegalovirus (CMV) and BK virus (BKV) infections did not occur at different frequencies in fast or slow TAC metabolizers and were uncommon after conversion to EVR.

**Figure 3:**
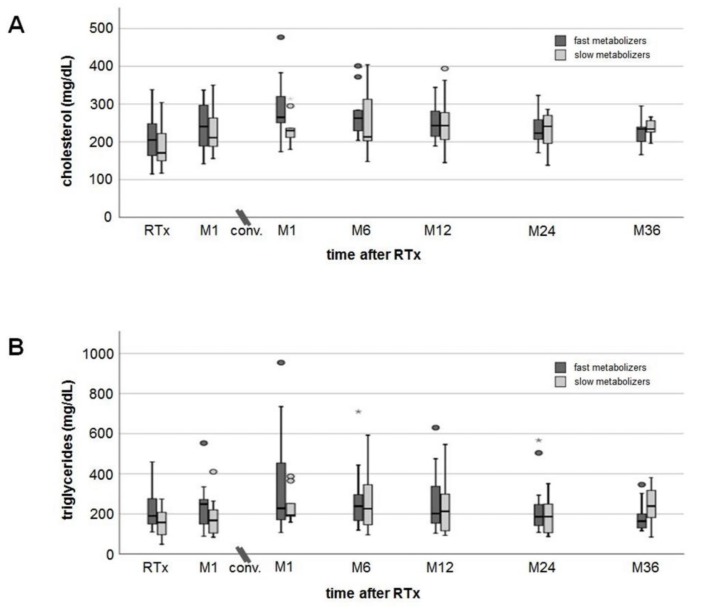

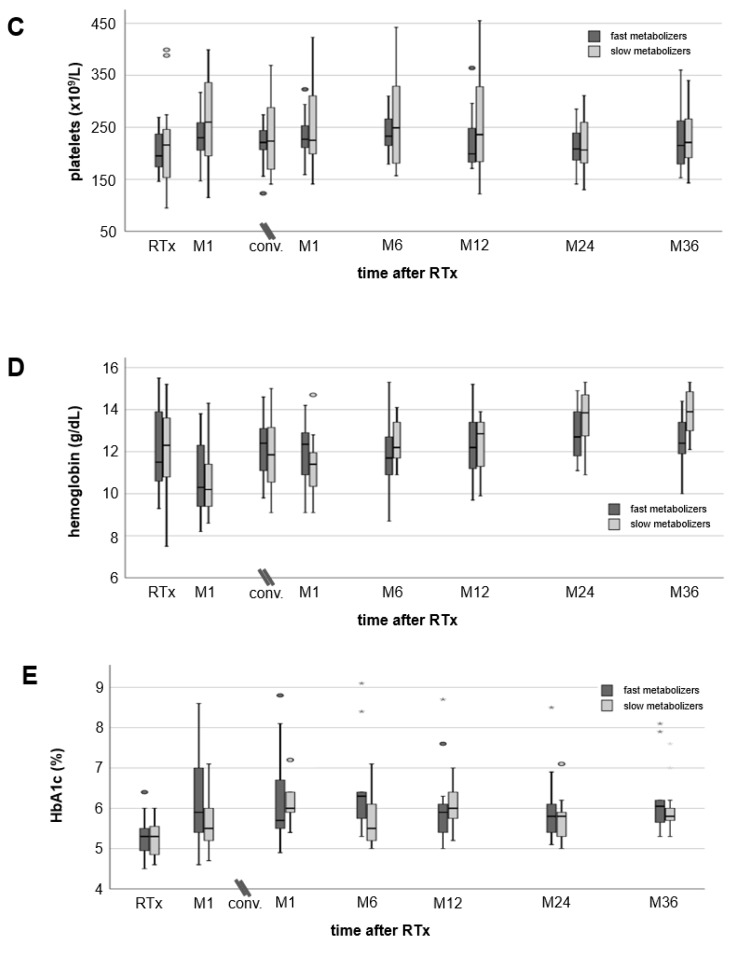
Courses of laboratory values. Cholesterol (**A**) and triglyceride (**B**) levels showed an increase after transplantation, but in a 36-month follow-up values decreased close to values measured at RTx (no noticeable differences between fast and slow metabolizers at any time). Mean platelets (**C**) and hemoglobin (**D**) remained in the normal range at all times without noticeable differences between the groups. Hemoglobin values dropped more than 1 g/dL at M1 after RTx, but had recovered already at the time of conversion from TAC to EVR (no noticeable differences between fast and slow metabolizers at all times). HbA1c levels (**E**) showed an increase one month after RTx without a relevant recovery during a 36-month follow-up after conversion. There were no noticeable differences in HbA1c values between the groups.

Cholesterol and triglycerides tended to be higher in fast than slow metabolizers (no noticeable differences, Figure 3A,B) and increased to a similar extent (approximately 20 mg/dL) in both groups after conversion to EVR. Platelets slightly increased after RTx but without differences between fast and slow metabolizers (Figure 3C). Hemoglobin levels decreased by 1 g/dL on average in both groups one month after RTx, but increased from 10.8 ± 1.7 g/dL (M1) to 12.5 ± 1.4 g/dL (M36 after conversion) in fast metabolizers and from 10.6 ± 1.6 g/dL (M1) to 13.9 ± 1.1 g/dL (M36 after conversion) in slow metabolizers (Figure 3D). Three years after conversion, hemoglobin levels were noticeably higher in slow metabolizers (*p* = 0.019). None of the RTx recipients needed erythropoiesis-stimulating agents. HbA1c levels increased slightly from 5.3% (4.5–6.4%) at RTx to 6.3% (5.3–9.1%) at M6 after conversion in fast metabolizers and from 5.3% (4.6–6.0%) at RTx to 5.5% (5.0–7.1%) at M6 in slow metabolizers (Figure 3E). HbA1c values decreased only slightly in both groups to a comparable extent until M36.

## 4. Discussion

The outcome of fast TAC metabolizers was shown to be inferior compared to the outcomes of slow TAC metabolizers when standard immunosuppression (immediate-release TAC, mycophenolate mofetil (MMF), and prednisolone) is used after RTx [4,5]. This finding was confirmed by others, even when higher C/D ratios were used for group definitions or when including patients receiving extended-release TAC [7,16]. In addition to increased rejection rates in patients with a low C/D ratio, increased rates of BK virus infection, CNI-related nephrotoxicity, and IF/TA were responsible for the lower eGFR of fast metabolizers [4,5,7,8,9,10]. In accordance with these data, Stegall et al. recently demonstrated in a large prospective cohort study using TAC-based immunosuppression that almost all kidney allografts have developed severe histological damage within ten years of RTx. However, the most frequently observed histological pathologies were arterial hyalinosis and glomerulosclerosis [17]. Both injuries can be linked to, e.g., CNI exposure [18]. Thus, CNI-induced nephrotoxicity remains a serious problem during CNI treatment [19]. Since only small case studies with patients who had CNI nephrotoxicity have investigated this conversion approach before and did not provide information regarding the TAC metabolism type of their patients, we herein investigated whether a conversion from TAC to EVR could be beneficial and safe for these patients [20,21].

In previous studies, we observed that as early as one month after RTx the kidney function of fast metabolizers is noticeably inferior to the kidney function of slow metabolizers [4]. Since TAC trough level and doses are usually higher within the first year after RTx and both can contribute to CNI nephrotoxicity, it is not surprising that CNI nephrotoxicity was the main reason for the conversion of TAC to EVR in our study cohort [10]. This disadvantage of fast metabolizers with respect to a lower eGFR persists over time and can still be observed to a large extent many years after Tx leading to inferior outcomes [5]. A comparable observation was made in liver transplanted patients [6]. However, in our present study, we were not able to show considerable advantages in fast metabolizers compared to slow metabolizers in relation to the eGFR after conversion of TAC to EVR. The change in eGFR from switching to M36 was similar in both, although a trend toward a higher increase in fast TAC metabolizers was observed. Two reasons might be responsible for this observation. First, the small number of cases could have masked the effect, especially when considering that a conversion of CNI to EVR usually leads to a small increase in eGRF. (This is independent of the type of TAC metabolism, although that has not been specifically studied before. For the first time, we present data relating to the C/D ratio before conversion.) [22] Notably, this effect may be more pronounced in cyclosporine-treated patients because cyclosporine A is a more potent vasoconstrictor than TAC [23,24]. Second, the time of the conversion could be relevant. Since renal function usually improves within the first year after transplantation due to the recovery from the transplant procedure and due to adaption of the kidney, these effects could also have an impact on the outcomes after conversion, since one may speculate that these processes might develop differently when using antiproliferative acting mechanistic target of rapamycin (mTOR)-inhibitors instead of CNIs [25,26,27]. In contrast to sirolimus-containing regimens [28,29], EVR-based immunosuppression was not found to lead to increased rates of delayed graft function or to poor results in terms of eGFR recovery after transplantation [30,31,32,33]. It was even postulated that progression of allograft fibrosis can be reduced by using mTOR-inhibition to down-regulate TGF-β signaling that is relevant for development of fibrosis [34]. However, even the large ELEVATE trial, which compared early conversion from TAC to EVR after RTx vs. CNI therapy, was not able to show differences between TAC- and EVR-treated patients in regards to the eGFR 12 months after RTx [35].

Nevertheless, we were able to show that conversion from TAC to EVR can improve eGFR even in RTx patients who had developed already CNI-induced side effects such as CNI nephrotoxcity—the main reason for conversion to EVR in our study. These data are in line with data from a small case series and a study showing reduced loss or even improvement of renal function after conversion to EVR in patients with CNI nephrotoxicity or chronic allograft nephropathy [20,21,36].

The overall rejection rate was low after conversion and not different between groups. No antibody-mediated rejection was observed until M36 and only one T-cell-mediated rejection occurred. Most importantly, we could not find any differences in (de novo) DSA. Based on our analyses at M36, class I DSA had occurred in only one patient (6%) of fast metabolizers. Due to previous transplantations, preformed Class II DSA were detectable in equal frequencies in both groups. The occurrence of de novo DSA apparently did not result in antibody-mediated rejection episodes within the three-year study period, as far as we know. However, rejections can occur later, as it is known from retrospective data that EVR-based regimens increase the risk of developing de novo DSA after RTx [37,38]. Interestingly, the prospective ELEVATE trial evaluated RTx patients with low immunological risk who were switched approximately three months after transplantation from CNI-based to EVR-based immunosuppression. One conclusion from the trial was that rejection rates in patients on the EVR-based regimen compared to patients receiving TAC had been higher; de novo DSA were not different between groups [35].

Consistent to previous data [27,35,39], after switching to EVR, we found no safety issues in either slow or fast TAC metabolizers (Table 3). However, others report high rates of adverse events and treatment discontinuation after conversion [30,40]. For example, the change in the lipid profile was as expected to occur for EVR, and showed no new safety concerns [35]. Notably, blood count and proteinuria even improved after conversion. It is known that mTOR-inhibition can be associated with a higher incidence of proteinuria compared to CNI treatment, an effect that is potentially dose-dependent [41,42,43]. However, it was suggested that especially late conversion promotes proteinuria. Our result is at least in line with the published results of others [44].

Of note, in this study, only one case of CMV infection occurred in fast metabolizers and no BKV infection after conversion. These data are consistent with randomized controlled trial data showing lower viral infection rates after switching to EVR [35].

The limitations of our study are the retrospective design and the limited sample size of our single-center study. However, we believe that our results are encouraging to design a prospective trial that can further evaluate our hypotheses.

In summary, we conclude from our data that selected RTx patients may benefit from a conversion from an immediate-release TAC-based immunosuppressive regimen to an EVR-based protocol to avoid further impair of kidney function associated with TAC treatment in these patients. This option could be especially interesting for patients who have already developed TAC-related adverse effects such as nephrotoxicity. Conversion to EVR is safe in selected slow and fast TAC metabolizers as the outcomes and the rate of adverse event did not noticeably differ between both TAC metabolizer types. However, these results must be confirmed in a prospective study.

## Figures and Tables

**Table 1:** Patient characteristics and immunosuppression.

	Fast Metabolizers (*n* = 17)	Slow Metabolizers (*n* = 17)	*p*-Values
Recipient Characteristics			
Sex (m/f)	11 (65%)/6 (35%)	10 (59%)/7 (41%)	1 ^a^
Age (year)	48.0 ± 15.7	54.6 ± 12.8	0.187 ^b^
Height (cm)	175.0 ± 10.7	171.4 ± 10.2	0.317 ^b^
Weight (kg)	79.0 ± 20.6	69.0 ± 11.9	0.095 ^b^
BMI (kg/m^2^)	24.7 (18.7–35.8)	22.3 (18.9–32.6)	0.114 ^c^
Transplant characteristics			
Number of RTx			
1	15 (88%)	13 (77%)	0.511 ^a^
2	2 (12%)	3 (18%)	
3	0	1 (6%)	
Living donor transplantation	6 (35%)	7 (41%)	1 ^a^
ABOi	0	2 (12%)	0.485 ^a^
ESP	1 (6%)	2 (12%)	1 ^a^
CIT (h)	6.8 (1.6–17.4)	5.5 (1.6–19.3)	0.838 ^c^
WIT (min)	35 (20–45)	30 (25–50)	0.858 ^c^
CMV risk			
Low	6 (35%)	2 (12%)	0.139 ^a^
Intermediate	7 (41%)	13 (77%)	
High	4 (24%)	2 (12%)	
Donor characteristics			
Donor sex (m/f)	10 (59%)/7 (41%)	6 (35%)/11 (66%)	0.303 ^a^
Donor age (year)	56.8 ± 8.8	57.4 ± 10.9	0.877 ^b^
Immunosuppression at M1			
TAC dose (mg)	12 (7–23)	7 (4–12)	<0.001 ^c^
TAC trough level (ng/mL)	8.5 (4.6–17.6)	10.0 (5.6–14.1)	0.208 ^c^
TAC C/D ratio (ng/mL*1/mg)	0.77 (0.40–1.00)	1.35 (1.05–2.56)	<0.001 ^b^
Prednisolone dose (mg)	20 (15–40)	20 (15–50)	0.422 ^c^
Mycophenolate mofetil dose (mg)	1000 (750–2000)	1000 (1000–2000)	0.501 ^c^

BMI, body mass index; RTx, renal transplantation; ABOi, ABO incompatible transplantation; ESP, European senior program; CIT, cold ischemia time; WIT, warm ischemia time; CMV, cytomegalovirus; TAC, tacrolimus; C/D, concentration/dose. Statistics: Variables are reported as absolute and relative frequencies, mean ± standard deviation or median (minimum–maximum). ^a^ Fisher’s exact test; ^b^ Welch’s t-test; ^c^ Mann–Whitney U-test.

**Table 2:** Reasons for the conversion to everolimus.

	Fast Metabolizers (*n* = 17)	Slow Metabolizers (*n* = 17)	*p*-Value
CNI-nephrotoxicity	13 (77%)	10 (59%)	0.277
chronic rejection	0	2 (12%)	
DGF	1 (6%)	2 (12%)	
NODAT	2 (12%)	0	
BKV-infection	0	1 (6%)	
neutropenia	0	1 (6%)	
neurotoxicity	0	1 (6%)	
study	1 (6%)	0	

CNI, calcineurin inhibitor; DGF, delayed graft function; NODAT, new onset diabetes mellitus after transplantation; BKV, BK virus. Statistics: Fisher’s exact test.

**Table 3:** Adverse events.

	Fast Metabolizers (*n* = 17)	Slow Metabolizers (*n* = 17)	*p*-Value
DGF	4 (24%)	5 (29%)	1 ^a^
Antibodies and rejection			
Preformed Class II DSA	1 (6%)	1 (6%)	1 ^a^
Class II DSA before conversion	1 (6%)	1 (6%)	1 ^a^
Class I DSA after conversion	1 (6%)	0	1 ^a^
BPAR before conversion to EVR			
AMR	1 (6%)	1 (6%)	0.490 ^b^
TCMR	1 (6%)	2 (12%)	
Combined AMR + TCMR	7 (41%)	3 (18%)	
BPAR after conversion to EVR			
AMR	0	0	0.485 ^a^
TCMR	2 (12%)	0	
Combined AMR + TCMR	0	1 (6%)	
Infections			
CMV infection before conversion	2 (12%)	4 (24%)	0.656 ^a^
CMV infection after conversion	1 (6%)	0	1 ^a^
BKV infection before conversion	2 (12%)	1 (6%)	1 ^a^
BKV infection after conversion	0	0	-
Death	0	1 (6%)	1 ^a^

DGF, delayed graft function; DSA, donor-specific antibody; BPAR, biopsy-proven acute rejection; AMR, antibody-mediated rejection; TCMR, T-cell mediated rejection; EVR, everolimus. Statistics: Adverse events are reported as absolute and relative frequencies. ^a^ Fisher’s exact test.
